# Generation of self-replicating airway organoids from the cave nectar bat *Eonycteris spelaea* as a model system for studying host–pathogen interactions in the bat airway epithelium

**DOI:** 10.1080/22221751.2022.2148561

**Published:** 2022-12-12

**Authors:** Louisa L.Y. Chan, Akshamal M. Gamage, Chee Wah Tan, Kai Sen Tan, Jing Liu, Douglas Jie Wen Tay, Randy Jee Hiang Foo, Laurent Rénia, De Yun Wang, Lin-Fa Wang

**Affiliations:** a Lee Kong Chian School of Medicine, Nanyang Technological University, Singapore; b Programme in Emerging Infectious Diseases, Duke-NUS Medical School, Singapore; c Department of Otolaryngology, Yong Loo Lin School of Medicine, National University of Singapore, Singapore; d Department of Microbiology and Immunology, Yong Loo Lin School of Medicine, National University of Singapore, Singapore; e Infectious Diseases Translational Research Program, Yong Loo Lin School of Medicine, National University of Singapore, Singapore; f Biosafety level 3 Core Facility, Yong Loo Lin School of Medicine, National University Health System, National University of Singapore, Singapore; g A*STAR Infectious Diseases Labs (A*STAR ID Labs), Agency for Science, Technology and Research (A*STAR), Singapore; h Singhealth Duke-NUS Global Health Institute, Singapore

**Keywords:** Bat, Chiroptera, *Eonycteris spelaea*, airway organoids, airway epithelial cells

## Abstract

Bats are reservoir hosts for various zoonotic viruses with pandemdic potential in humans and livestock. *In vitro* systems for studying bat host–pathogen interactions are of significant interest. Here, we establish protocols to generate bat airway organoids (AOs) and airway epithelial cells differentiated at the air–liquid interface (ALI-AECs) from tracheal tissues of the cave-nectar bat *Eonycteris spelaea*. In particular, we describe steps which enable laboratories that do not have access to live bats to perform extended experimental work upon procuring an initial batch of bat primary airway tissue. Complete mucociliary differentiation required treatment with IL-13. *E. spelaea* ALI-AECs supported productive infection with PRV3M, an orthoreovirus for which Pteropodid bats are considered the reservoir species. However, these ALI-AECs did not support SARS-CoV-2 infection, despite *E. spelaea* ACE2 receptor being capable of mediating SARS-CoV-2 spike pseudovirus entry. This work provides critical model systems for assessing bat species-specific virus susceptibility and the reservoir likelihood for emerging infectious agents.

## Introduction

Airway organoids (AOs) and airway epithelial cells differentiated at the air–liquid interface (ALI-AECs) are emerging as important *in vitro* tools for studying host–pathogen interactions, as they better model the complex cellular heterogeneity as well as the spatial arrangement of the airway epithelium compared to cell lines [1,2]. AOs are multicellular, self-assembling, and self-replicating structures derived from adult progenitor/stem cells obtained from primary airway tissue. Pseudostratified AOs displaying mucociliary differentiation can be expanded long-term (>1 year) in differentiation media containing a milieu of growth factors and compounds without sacrificing the original cellular, molecular and genetic characteristics [3]. ALI differentiated airway epithelial cells can be similarly generated from adult stem cells obtained from primary tissue, and also possess a heterogeneous cellular composition resembling that of airway epithelia *in-vivo*. Over the past decade, several breakthroughs have established and optimized protocols for the generation of AOs and epithelial ALI cultures from human and mouse airway tissues. However, protocols for establishing these models in other non-typical species are rare and not well characterized.

Bats are hosts to a large diversity of viruses and are a reservoir host for, or associated with, several zoonotic pathogens including MERS-CoV, SARS-CoV, rabies, Nipah, and Ebola viruses [4]. Therefore, establishing AOs and epithelial ALI cultures from bat species provide a useful tool for investigating the bat response to infection at the mucosal surface. However, Chiropteran research work suffers from several challenges, including the lack of bat species-specific commercial reagents such as recombinant cytokines and antibodies. There is also a paucity of access to primary material by most laboratories, due to the presence of only a limited number of captive breeding colonies for research purposes [5]. *Eonycteris spelaea* is a nectarivorous bat species in the Pteropodidae family, within the Yangochiroptera (megabat) lineage. Work done by our group and others have helped to establish *E. spelaea* as a pertinent model species for bat research. Positive attributes of *E. spelaea* as a model bat species include its ability to breed readily in captivity, small size for ease of handling, and a draft genome available [6,7]. On-going work to generate an improved reference-quality genome and a single-cell transcriptome atlas of key *E. spelaea* organs and tissues would further facilitate research work in this species. *E. spelaea* has been documented to harbour various zoonotic viruses, included coronaviruses [8,9], Pteropine orthoreviruses [10], and astroviruses [11]. In this study, we establish protocols for the propagation of AOs derived from *E. spelaea* primary tracheal tissue, followed by their ALI differentiation as a suitable model system for studying the bat response to viral infection.

## Materials and methods

### Bat tissue processing and isolation of bat airway epithelial cells (bAECs)

*E. spelaea* bats used in this study were part of a captive breeding colony and handled according to protocols approved by the SingHealth Institutional Animal Use and Ethics Committee (IACUC number 2020/SHS/1582). Tracheal tissue were cut into approximately 1 mm^3^ pieces and rinsed with Hank's Balanced Salt Solution (HBSS) supplemented with 2.5 µg/mL amphotericin B, 50 µg/ml gentamicin and 2% P/S. Tissue fragments were incubated with 20 mg/ml protease type XIV in RPMI 1640 supplemented with 2.5 µg/mL amphotericin B, 50 µg/ml gentamicin, and 2% P/S overnight with rotation at 4°C. Digested tissues were filtered through a 100 µM cell strainer and cell clumps retained on the strainer were dissociated with a syringe plunger. Cells were centrifuged and the pellet was resuspended in RPMI 1640 with 10% FBS to inhibit digestion. Cells were washed with RPMI 1640 twice and plated on human collagen IV pre-coated 6-well plate and cultured in B/D expansion medium (Supplementary Table 1) to grow as a monolayer. Medium was refreshed every two days until confluent. Undifferentiated cells derived from digestion of primary tissue were also stored as frozen stocks in CryoStor® CS10 cell freezing medium (StemCell technologies) for future use.

### Bat ALI-AEC culture

Once the stem/progenitor cells formed a confluent monolayer, 2 × 10^5^ cells were seeded on the apical chamber of a 24-well Transwell (Corning Transwell 3470) pre-coated with 30 µg/ml PureCol (Advanced BioMatrix). Cells seeded on Transwells were first cultured in submerged phase. Once the cell layer on the Transwell membrane was observed to be intact and fully confluent (2-4 days post-seeding), the medium on the apical chamber was removed and the cells were cultured at ALI thereafter. Basolateral medium was refreshed twice a week and the cells were differentiated for 18 days in ALI-Diff medium (Supplementary Table 2) and subsequently differentiated into airway organoids (AOs). To promote goblet cell differentiation, bAECs cultured for 7 days at ALI were treated with 10 ng/ml human Inteleukin-13 (IL-13, StemCell technologies) for an additional 14 days before cell infection or other assays. Frozen stocks of ALI-AECs dissociated into single cells were stored in CryoStor^®^ CS10 cell freezing medium (StemCell technologies).

### Bat AO differentiation

ALI-AECs differentiated for 18 days were detached from Transwells with TrypLE and resuspended in Matrigel culturing in Airway Organoid (AO) medium (Supplementary Table 3) supplemented with 25% R-spondin-1 conditioned medium, 25 ng/ml FGF7 and 100 ng/ml FGF10 for 3D expansion. Cells were expanded and formed spheroid structures in Matrigel. At day 5 of expansion, spheroids were passaged onto the apical chamber of a 12-well insert for differentiation at ALI for an additional 4–6 weeks. Medium was refreshed twice a week using AO medium with 5 ng/ml FGF7 and 20 ng/ml FGF10 until the organoids were well-differentiated. Once AOs were mature, they were frozen in CryoStor^®^ CS10 cell freezing medium (StemCell technologies) for long-term storage.

### Conversion from organoids to ALI

To convert mature AOs into ALI-AEC culture format, organoids were dissociated into small fragments or single cells with TrypLE express and seeded on the apical chamber of a 24-well Transwell pre-coated with 30 µg/ml PureCol with ALI-Diff medium. Cells were maintained in sub-merged manner until confluent monolayer was achieved after 2–4 days. Cells were then cultured at ALI and basolateral medium was refreshed twice a week until day 21 of differentiation.

### IFA staining of organoids and ALI cultures

ALI cultures and intact organoids were fixed with 4% paraformaldehyde for 30 mins at room temperature followed by washing with PBS and permeabilized with 0.2% Triton X-100 in PBS for 30 min. This was followed by blocking with staining buffer (1% BSA, 0.2% Triton X-100 in PBS) for 1 h. Cells were next incubated with primary antibodies with cross-reactivity to *E. spelaea* including mouse anti-acetylated α-tubulin (6-11B-1) (Cat#T7451, Sigma, USA), mouse anti-MUC5AC (45M1) (Cat#MA5-12178, ThermoFisher Scientific, USA) and rabbit anti-TP63 (EPR5701) (Cat#ab124762, Abcam, UK) diluted in staining buffer for 3 h at room temperature, followed by washing three times with PBS. AlexaFluor 488 or 594-labelled secondary antibodies (Thermofisher Scientific, USA), phalloidin, and 4′,6-diamidino-2-phenylindole (DAPI) (Abcam, UK) were incubated with for 1 h at room temperature. After staining, membranes with cells attached were detached from the Transwell inserts. Membranes or organoids were mounted on glass slides with ProLong™ Glass antifade mountant (ThermoFisher Scientific, USA) and covered with glass coverslips. Images were captured using a Zeiss LSM 800 confocal microscope and processed using ZEN Image analysis software (Zeiss).

### Alcian blue staining

ALI cultures were fixed with 4% PFA for 15 min at room temperature, embedded in paraffin, and sectioned at a thickness of 4 µm. Alcian blue staining was then carried out (Alcian Blue pH2.5 Periodic Acid-Schiff stain kit, Thermofisher Scientific, USA). The sections were deparaffinized and rehydrated to deionized water. Then placed slides in alcian blue pH 2.5 stain solution for 30 min at room temperature. Rinsed sections and then placed sections in periodic acid solution (0.5%) for 5 min at room temperature. Rinsed sections and stained in Schiff reagent for 15 min. Rinsed and dehydrated sections then mounted with mounting medium.

### RNA extraction, cDNA synthesis, and quantification of gene expression by quantitative PCR (qPCR)

Total RNA was extracted using the RNeasy Plus Mini Kit (Qiagen, Germany) according to the manufacturer's instructions. Reverse transcription was then performed using the PrimeScript RT Reagent Kit (Takara Bio, USA) to generate cDNA under the following conditions: 37°C for 15 mins, 85°C for 5s followed by 4°C incubation. Diluted cDNA samples were used for real-time PCR with the SensiFAST SYBR No-ROX Kit (Bioline), on a CFX96 Touch Real-Time PCR Detection System (Bio-Rad) with the cycling parameters: 95°C for 5 min, followed by 45 cycles of 95°C for 10 s, 60°C for 30 s and plate read step. *E. spelaea* real-time PCR primer sequences used in this study are listed in Supplementary Table 4. Target gene expression was normalized to the geometric mean of three house-keeping genes (β actin, SNPD3 and RPL4).

### PRV3M and SARS-CoV-2 infection, virus titration, and RNA extraction

On the day prior to infection, the apical surface of IL-13 treated ALI-AECs was washed once with 1x Dulbecco's PBS (dPBS) to remove excess apical secretions, and the basolateral media changed to ALI-Diff medium without IL-13. ALI-AECs were infected with PRV3M at an MOI of 0.01, or SARS-CoV-2 Wuhan-Hu-1 strain or Omicron variant at a MOI of 0.1. Stock virus was diluted in 1x dPBS, and 100 µl of virus was added to the apical surface. After incubation at 37°C for 60 min, apical inoculum was removed and stored for titration. The apical surface was washed once with 1x dPBS, and basolateral media replaced with fresh ALI-Diff medium, and the cells placed back in the incubator. At indicated times post-infection, apical surface was rinsed with 100 µl of dPBS. The apical washes were used for virus titration. For PRV3M virus titration, 10-fold serial dilutions were added onto HEK293T cells seeded in 96-well plates. The plates were observed for cytopathic effect (CPE) after 5 days on incubation, and the 50% tissue culture infective dose (TCID_50_) calculated. RNA was extracted from uninfected, or PRV3M infected ALI-AECs by lysing cells in TRK buffer, using an E.Z.N.A.® Total RNA Kit I (Omega Bio-tek) according to manufacturer's instructions. The cDNA was synthesized using a QuantiTect Reverse Transcription Kit (Qiagen) according to manufacturer's instructions, and real-time PCR performed as described above. For SARS-CoV-2 virus titration, 10-fold serial dilutions were added onto A549 cells over-expressing ACE2, and incubated at 37°C for 1 h, after which the media was removed and overlaid with plaque medium containing 0.5% carboxylmethylcellulose and 0.5% avicel. At 3 dpi, cells were fixed with 4% formaldehyde, stained with 0.2% crystal violet, and plaques enumerated.

### Giemsa staining

Uninfected, or PRV3M infected AECs were fixed in 100% methanol for 15 min, and air dried. The inserts were stained with modified Giemsa stain (Sigma) for 45 minutes and rinsed with distilled water. Detached transmembranes were air dried and imaged on an Axio Lab A1 microscope (Carl Zeiss) under bright field filter.

### E. spelae ACE2 cloning and SARS-CoV-2 spike pseudovirus entry assay

*E. spelaea* ACE2 was amplified from kidney tissue cDNA using the primers Age1_Kz_F TAAGCAACCGGTCACCATGTCAGGCTCTTTCTGGCT and No_Stop_Pac1_R GCTGACTTAATTAAAAATGAAGTCTGAACATCATCA (underlined sequences correspond to the ACE2 nucleotide sequence at termini) and cloned into Age1 and Pac1 digested pLenti plasmid with an in-frame C-terminus FLAG tag. HEK293T cells were seeded on poly-L-lysine coated 96 well plates and transfected with 200 ng/well of plasmid expressing either human or *E. spelaea* ACE2-FLAG constructs. Control wells were not transfected with plasmid. After 24 h of transfection, media was removed and replaced with 50 µl of SARS-CoV-2 Omicron spike pseudovirus (neat and 10x dilution in media) generated as previously described [12]. After a further 24 h of incubation, 50 µl of ONE-Glo luciferase substrate (Promega) was added and the luminescence signal was measured using the Cytation 5 microplate reader (BioTek) with Gen5 software version 3.10 [12]. Pseudovirus entry was expressed as fold change in luminescence between ACE2-FLAG transfected cells vs untransfected controls.

## Results

### Generation of bat AO and ALI-AECs from Eonycteris spelaea

**Figure 1.**
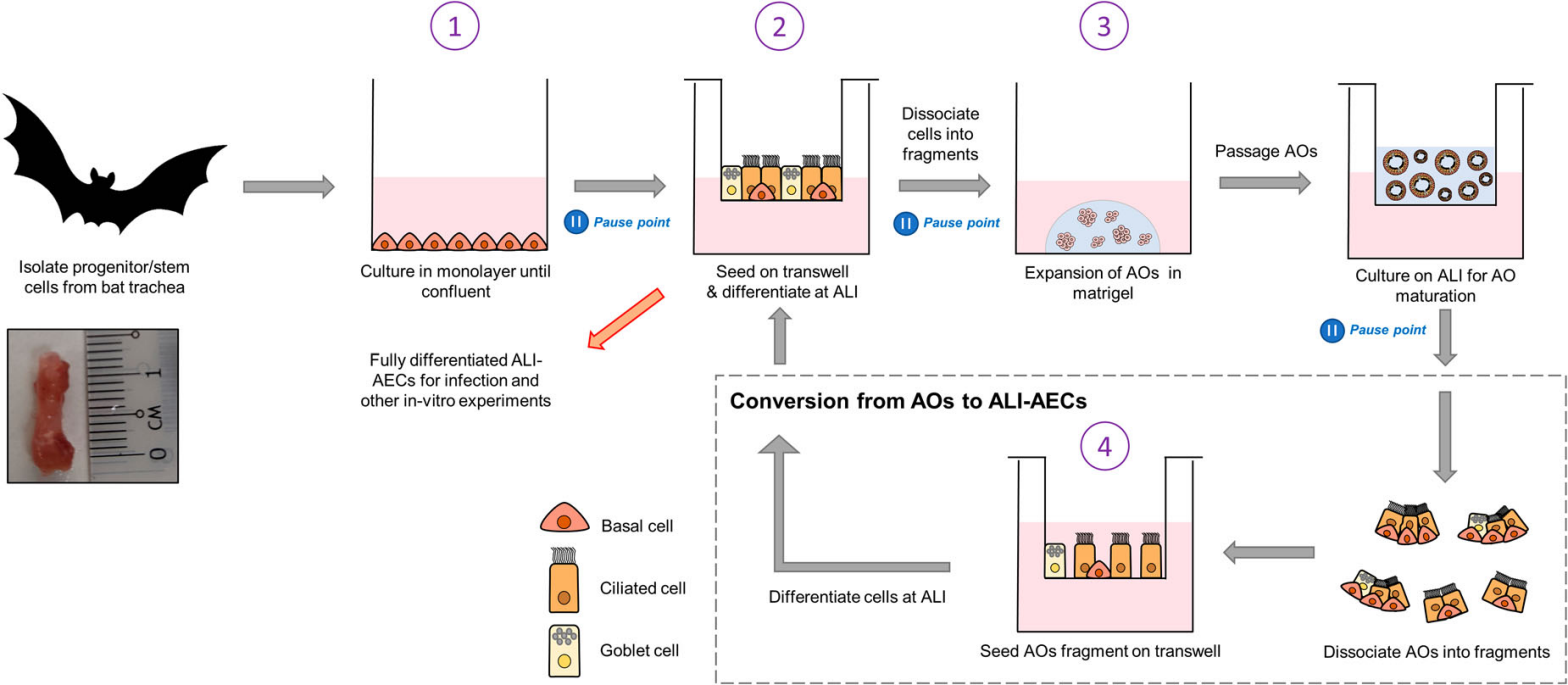
Establishment of AOs and ALI-AECs from *Eonycteris spelaea*. Schematic illustration of the isolation and expansion of bat airway progenitor/stem cells (Stage 1), Transwell differentiation into ALI-AECs (State 2), mature AO differentiation (Stage 3), and the recursive generation of ALI-AECs from AOs (Stage 4). Inset: Bat trachea tissue, with 1 cm marker for scale. Pause points: The culture protocol can be halted at the indicated points, and single-cell suspensions frozen in liquid nitrogen for revival later.

Tracheal tissues were used as an easily accessible source of upper-airway tissue from *E. spelaea*. We next established a 4-stage protocol for continuously expanding bat AECs. In the first stage, progenitor/stem cells are extracted from bat tracheal tissue and expanded as a monolayer. They are then differentiated into ALI-AECs on Transwell inserts (Stage 2). Differentiated AECs are fragmented and expanded as AOs in Matrigel media (Stage 3). Differentiated AOs can be passaged routinely every 2–3 weeks, consistent with human and mouse AO protocols. For infection and other *in vitro* studies needing access to the apical surface, AOs were fragmented, seeded on Transwell membranes and cultured into polarized AECs after growing at the air–liquid interface (Stage 4). At the end of stages 1, 2, and 3 (indicated by pause points in Figure 1), cells can be dissociated, re-suspended in freezing media, aliquoted in vials and stored in liquid nitrogen for later work.

### Characterization of differentiated bat airway cultures

**Figure 2.**
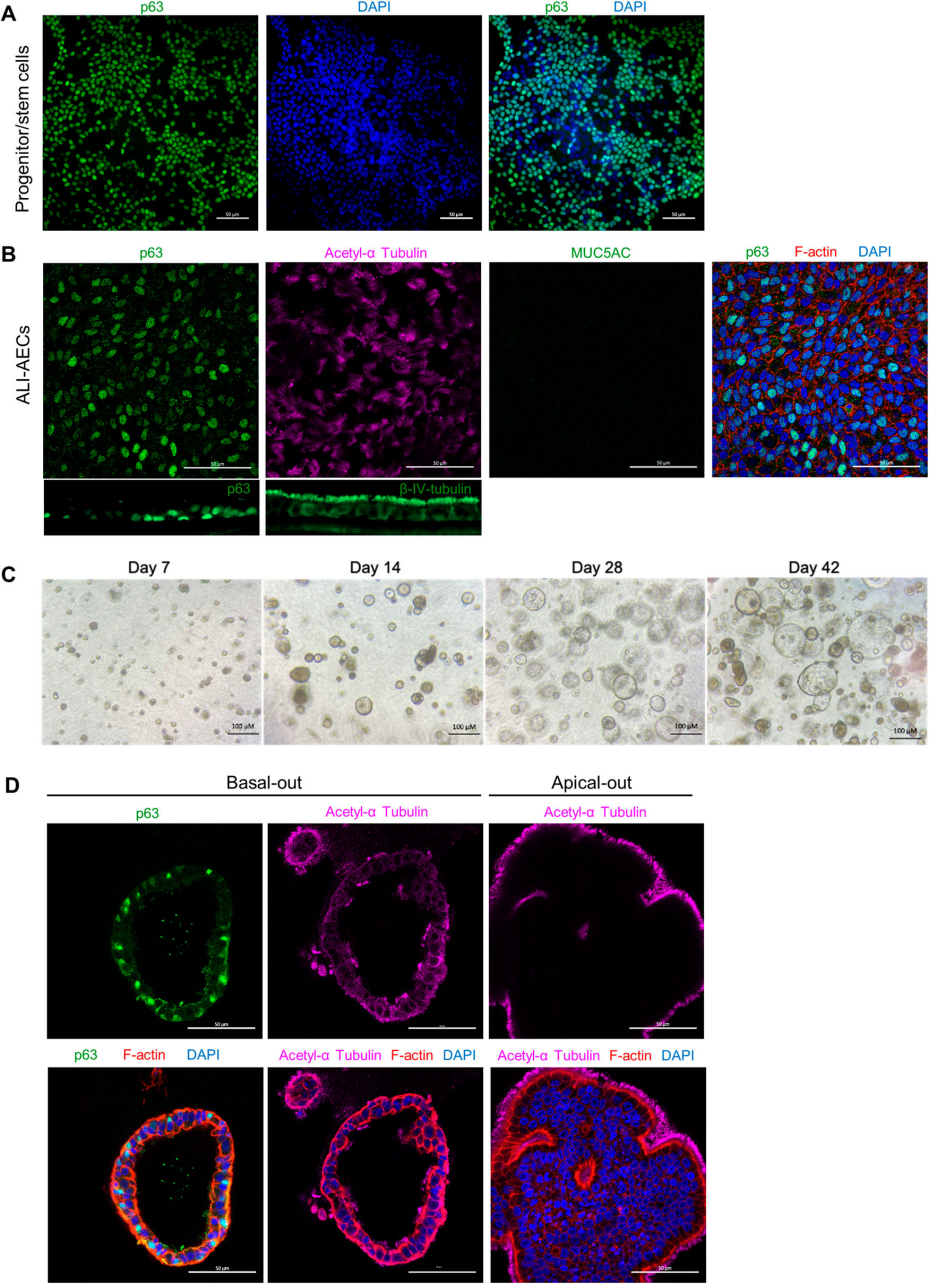
Characterization of bat AO and ALI-AECs. (A) Immunofluorescence staining of progenitor/stem cells for basal cell marker P63 (green) and cell nuclei (DAPI, blue). Scale bar  = 50 μΜ. (B) Immunofluorescence staining of ALI-AECs for P63 (green), ciliated cell marker acetylated-α-tubulin (purple) or β-IV-tubulin (green, for cross-section image), and goblet cell marker MUC5AC (green, absent). Cell nuclei and F-actin are counterstained with DAPI (blue) and phalloidin (red). Scale bar  = 50 μΜ. (C) Bright field images of bat organoids at day 7, 14, 28 and 42 of differentiation. Scale bar  = 100 μΜ. (D) Differentiated bat organoids in basal-out or apical-out orientation stained for P63 (green), acetylated-α-tubulin (purple), F-actin (red) and nucleus (blue). Scale bar  = 50 μΜ.

The *E. spelaea* (cave nectar bat) genome contained homologues for several markers which have been used in the literature to identify epithelial cell types in human AECs (Supplementary Table 5), Bat progenitor/stem cells expanded as a monolayer were positive for P63, a basal cell marker (Figure 2A). When the cells were differentiated at ALI, a polarized, pseudostratified airway epithelium containing P63 + basal and acetylated α-tubulin+/ β-IV-tubulin + multi-ciliated cells were observed. However, MUC5AC + goblet cells were not observed (Figure 2B). To verify that anti-MUC5AC antibody was capable of binding its cognate target on bat goblet cells, a cross section of the bat nasal septum was stained with the same antibody (Figure S1). Abundant goblet cells staining MUC5AC + were observed, along with β-IV-tubulin + multi-ciliated cells (Figure S1).

**Figure 3.**
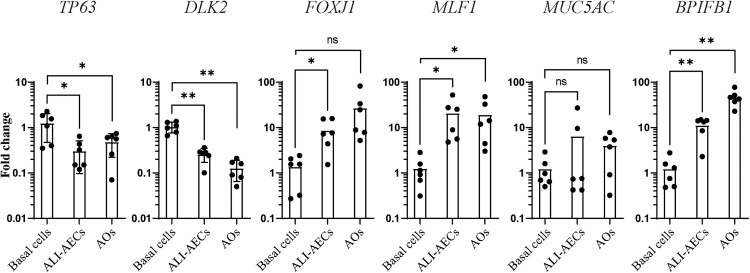
Gene expression changes during bat airway epithelial cell differentiation. qRT-PCR analysis of progenitor/stem cells, ALI-AECs and AOs for basal cell markers *P63* and *DLK2*, ciliated cell markers *FOXJ1* and *MLF1* and goblet cell markers *MUC5AC* and *BPIFB1*. *n* = 6 biologically independent bat donors are illustrated. Each dot represents airway culture derived from a different bat donor. Each bar represents the mean. * indicates Student's t-test *p*-value <0.05, ** indicates Student's t-test *p*-value <0.01 for the indicated comparisons.

Cells detached from ALI culture were next cultured in Matrigel to form spheroids (Figure 2C). As the differentiation of these spheroids progressed in culture, they were observed to increase in size, with formation of lumens within the pseudostratified airway epithelium (Figure 2C–D). Similarly to ALI-AECs, P63 + basal cells and acetylated α-tubulin + ciliated cells were the only observable cell types in the organoids (Figure 2D). qPCR analysis revealed that expression of basal cell markers, *P63* and *DLK2* decreased during the differentiation of ALI-AECs and AOs from progenitor/stem cells (Figure 3). Ciliated cell markers *FOXJ1* and *MLF1* as well as the goblet cell marker *BPIFB1* were generally observed to increase in expression from the differentiated cultures (Figure 3). *MUC5AC* expression did not significantly increase during differentiation, consistent with MUC5AC^+^ goblet cells not identified in the prior immunofluorescence staining.

### Treatment with human IL-13 induces goblet cell differentiation

**Figure 4.**
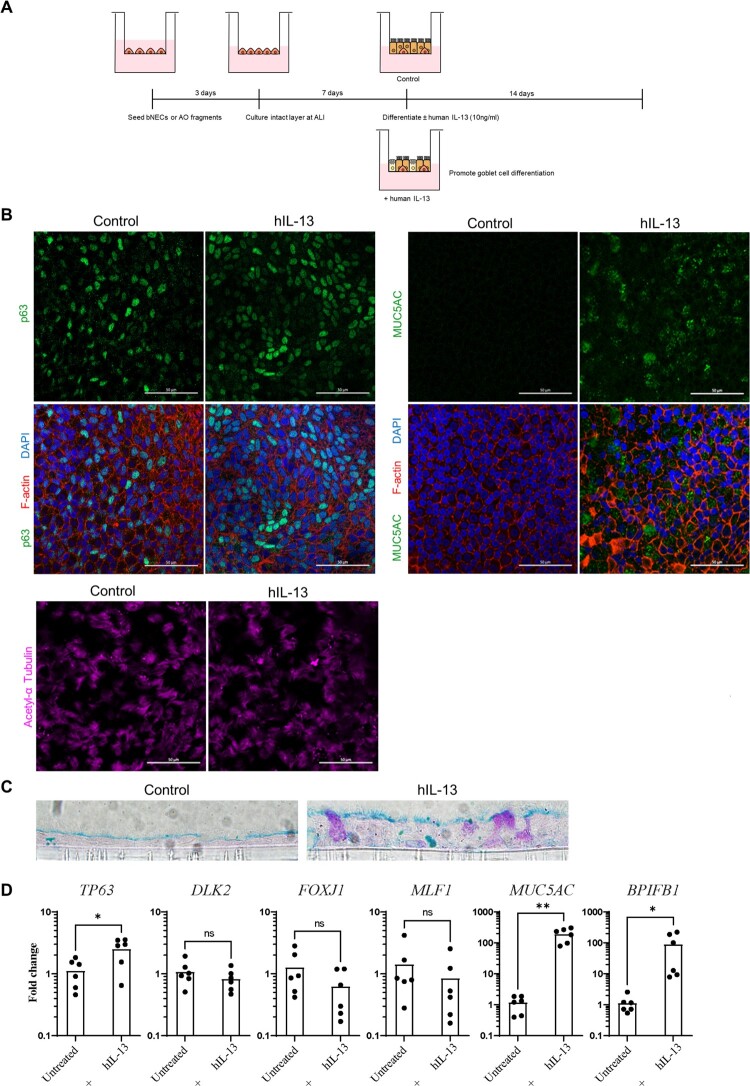
Human IL-13 (hIL-13) treatment promotes goblet cell differentiation in ALI-AECs. (A) Schematic representation of culture protocol with and without hIL-13 (10 ng/ml) treatment. (B) Untreated or hIL-13 treated ALI-AECs stained for P63 (green), acetylated-α-tubulin (purple), F-actin (red) and nucleus (blue). Scale bar  = 50 μΜ. (C) Alcian blue staining of untreated or hIL-13 treated ALI-AEC cross sections. Representative results from two individual bat derived AECs are shown. Scale bar  = 10 μΜ (D) qRT-PCR analysis of untreated or hIL-13 treated ALI-AECs for indicated marker genes. *n* = 6 biologically independent bat donors are illustrated. Each dot represents airway culture derived from a different bat donor. Each bar represents the mean. * indicates Student's t-test *p*-value <0.05, ** indicates Student's t-test *p*-value <0.01 for the indicated comparison.

We next explored whether IL-13 treatment could drive goblet cell differentiation in ALI-AECs. IL-13 has been well-established to induce mucus production and goblet cell hyperplasia in human primary airway cultures. Treatment with 10 ng/ml of recombinant human IL-13 was introduced at day 7 of ALI culture and continued for an additional 14 days (Figure 4A). IL-13 treated ALI-AECs demonstrated MUC5AC + goblet cells, while also staining for the presence of P63 + basal cells and α-tubulin + ciliated cells (Figure 4B). To further verify the presence of mature goblet cells in IL-13-treated bat ALI-AECs, cross-sections were subjected to Alcian blue staining, which stain for acidic mucins. Alcian blue positive cells were only observed upon IL-13 treatment of bat ALI-AECs (Figure 4C). Consistent with the immunofluorescence staining data, IL-13 treatment potently upregulated average expression of goblet cell markers *MUC5AC* and *BPIFB1* by 160- and 78-fold, respectively (Figure 4D).

### E. spelaea ALI-AECs are susceptible to PRV3M but not SARS-CoV-2

**Figure 5.**
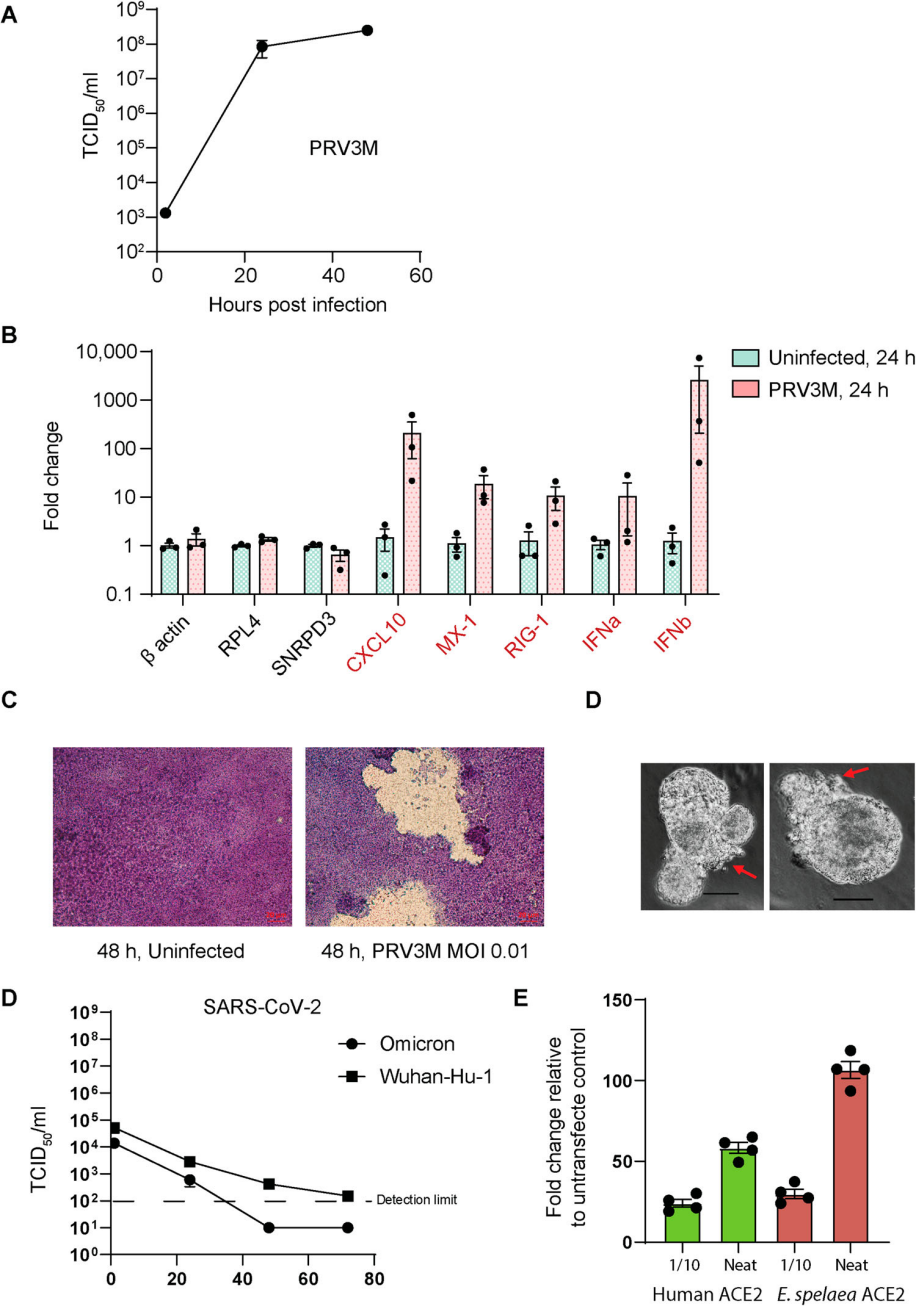
hIL-13 treated ALI-AECs are susceptible to PRV3M (Melaka virus) infection but not to SARS-CoV-2 infection. (A) Viral replication kinetics of PRV3M released from the apical surface of infected ALI-AECs at 1, 24 and 48 hpi. Each dot represents data from a biologically independent bat donor, *n* = 3. Data represented as mean ± SEM. (B) qRT-PCR analysis of uninfected or PRV3M-infected ALI-AECs at 24 hpi for housekeeping genes β*-actin*, *RPL4* and *SNRPD3*, Type I IFNs (*IFNα* and *IFNβ*) and ISGs *CXCL10*, *MX-1* and *RIG-I*. Each dot represents data from a biologically independent bat donor, *n* = 3. Data are presented as mean ± SEM. (C) Bright field images of Giemsa stained TransWell membranes of uninfected or PRV3M-infected ALI-AECs. Scale bar  = 20 μΜ. (D) Bright field images of PRV3M infected organoids at 48 h post-infection. Red arrows indicate damage to organoid epithelial structure. Scale bar  = 20 μΜ. (E) Viral replication kinetics of indicated SARS-CoV-2 strains released from the apical surface of infected AECs at 1, 24, 48 and 72 hpi. Each dot represents data from a biologically independent bat donor, *n* = 3. (F) Fold change in luminescence upon infection of HEK293T cells over-expressing either human or *E. spelaea* ACE2-FLAG with SARS-CoV-2 spike pseudovirus, relative to reading from untransfected cells.

Lastly, we explored the use of bat ALI-AECs for studying zoonotic virus infection. The Pteropine orthoreovirus PRV3M is a double-stranded RNA virus for which Pteropodid bats are considered the reservoir hosts [13]. PRV3M was capable of rapidly infecting *E. spelaea* ALI-AECs, reaching a titer of approximately 8.4 × 10^7^ TCID_50_/ml within 24 h post infection (hpi), upon infection at an MOI of 0.01 (Figure 5A). This was accompanied by the transcriptional up-regulation of Type I IFN genes *IFNA* and *IFNB*, as well as the interferon-stimulated genes *CXCL10*, *MX1,* and *RIG-1* (Figure 5B). Extensive damage to the ALI-AEC structure was visible within 48 h of infection (Figure 5C), caused by the disintegrated of syncytial structures. Similarly, infection of bat AOs with PRV3M resulted in a visible cytopathic effect on the epithelial cell structure within 48 h of infection (Figure 5D). In contrast, SARS-COV-2 Wuhan-Hu-1 and Omicron variants failed to establish productive infection within *E. spelaea* ALI-AECs upon infection at an MOI of 0.1, as followed for up to 72 h post-infection (Figure 5E). However, both *E. spelaea* and human ACE2 were capable of mediating entry of SARS-CoV-2 spike pseudoviruses (Figure 5F), indicating that the failure for SARS-CoV-2 infection was not due to receptor incompatibility with the *E. spelaea* ACE2 homologue.

## Discussion

Recent studies reported the generation of intestinal organoids from Chinese horseshoe bat, *Rhinolophus sinicus* [14] and Leschenault's rousette, *Rousettus leschenaultia* [15]. To our knowledge, this is the first study to establish a sustainable approach for the propagation of bat airway organoids, and their conversion into ALI-AECs to aid infection studies. Together, these studies will provide powerful new tools for investigating host–pathogen interactions at the bat mucosal surface. ALI-AECs cultured on standardized Transwell inserts have a more readily accessible apical surface with direct exposure of luminal cells to pathogen of interest that provide more replicable conditions for infection experiments compared to organoids embedded in Matrigel media [16]. However, unlike the generation of AOs, epithelial ALI culture is a single-use experimental procedure, as the epithelial structures grown on Transwell inserts are not amenable for further passaging and onset of cellular senescence after prolonged culturing. This protocol incorporates the advantages of both approaches, along with the capacity of long-term expansion and cryopreservation, enabling more widespread adoption of bat airway organoid work by research groups which do not have access to a captive-bred bat colony or primary tissue on a regular basis.

We report that although a pseudostratified epithelium with the presence of basal and ciliated cells can be differentiated under similar chemical conditions used for human AECs, additional IL-13 treatment is required for the generation of mature goblet cells for *E. spelaea* to better mimic its airway microenvironment. IL-13 has been well documented to increase goblet cell differentiation in humans [17] and rodent models [18], mediated by direct signaling via the IL-13 receptor expressed on airway epithelial cells [19]. Recombinant human IL-13 induced a similar phenotype in bat AECs, indicating conserved IL-13 mediated goblet cell differentiation mechanisms in *E. spelaea* to that observed in humans and rodents. IL-13 treatment also had a small but significant increase in TP63 expression. IL-13 has been previously reported to modulate P63 expression during human keratinocyte differentiation [20]. Further work is warranted to identify why the basal differentiation medium was not sufficient to induce goblet cell differentiation in bat AECs. More broadly, bat AOs and ALI-AECs provide an additional mammalian model system for studying the differentiation trajectories of various epithelial cell types during mucociliary differentiation, and the identification of conserved and unique differentiation pathways between bats and other mammals.

These *in-vitro* systems can also be used for the isolation and propagation of zoonotic viruses from their respective bat hosts, for downstream experimental characterization. As the mucosal surface is a common route of infection for pathogens, these AOs and ALI-AECs are likely to harbour the requisite receptors and host entry factors to facilitate viral replication and isolation. For example, human noroviruses, the most common cause of epidemic and sporadic acute gastroenteritis, only recently became possible to be cultured using a human intestinal organoid-derived epithelial monolayer [21,22]. Of particular interest, recent bat surveillance studies have identified various beta coronaviruses in *E. spelaea* and other closely related Pteropodid bats [23–26], for which virus isolation or receptor characterization studies are still lacking.

Lastly, we show that *E. spelaea* ALI-AECs are not susceptible to SARS-CoV-2 infection, but show robust infection with PRV3M, a zoonotic virus frequently isolated from Pteropodid bats. To date, ACE2 binding sarbecoviruses have been primarily detected from *Rhinolophus* species [27], with limited evidence of other bat families hosting these viruses. Our findings are consistent with these environmental sampling data. Similarly, it was observed that intestinal organoids from another Pteropodid bat, *R. leschenaultia*, were not susceptible to SARS-CoV-2 [15], while intestinal organoids from a *Rhinolophus* species readily supported SARS-CoV-2 replication [28]. The observed lack of susceptibility to SARS-CoV-2 is despite the *E. spelaea* ACE2 receptor homologue mediating SARS-CoV-2 spike pseudovirus entry. This demonstrates that factors additional to ACE2 receptor compatibility, including ACE2 protein expression, protease expression, and host antiviral responses are important in determining host susceptibility to SARS-CoV-2. Bat airway culture would therefore provide a useful and timely model to determine the likelihood of zoonotic spillover and potential reservoir capabilities from different bat species for emerging infectious diseases.

## Author contributions

Conceptualization: LLYC, AMG, TKS, LJ, WDY and WLF. Investigation & methodology: LLYC, AMG, TKS, TCW, LJ, DJWT, RJHF. Writing: LLYC, AMG, TKS, TCW, LJ. Editing: LLYC, AMG, TKS, TCW, LJ, LR. Funding acquisition & supervision: WLF, WDY, LR. All authors have read and agreed to the final version of the manuscript.

## Supplementary Material

Supplemental Material

Supplemental Material
