# Supplementary figures and images for: Generation of self-replicating airway organoids from the cave nectar bat Eonycteris spelaea as a model system for studying host–pathogen interactions in the bat airway epithelium
Source: Emerg Microbes Infect. 2022 Dec 12;12(1):e2148561. doi: 10.1080/22221751.2022.2148561 (PMC9754017; doi:10.1080/22221751.2022.2148561)

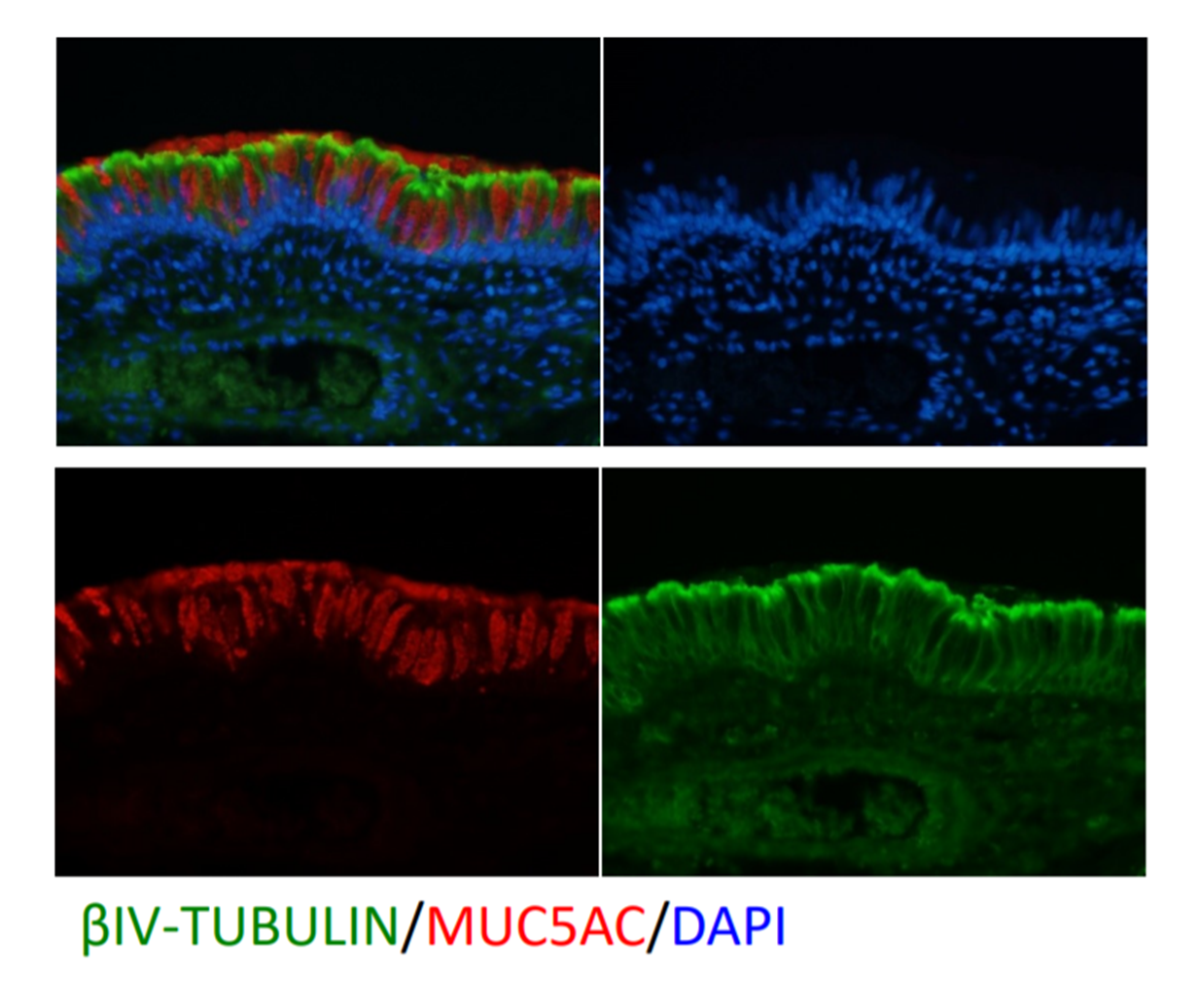

Supplement: Supplemental Material [file TEMI_A_2148561_SM6084.zip › Fig S1.tif]

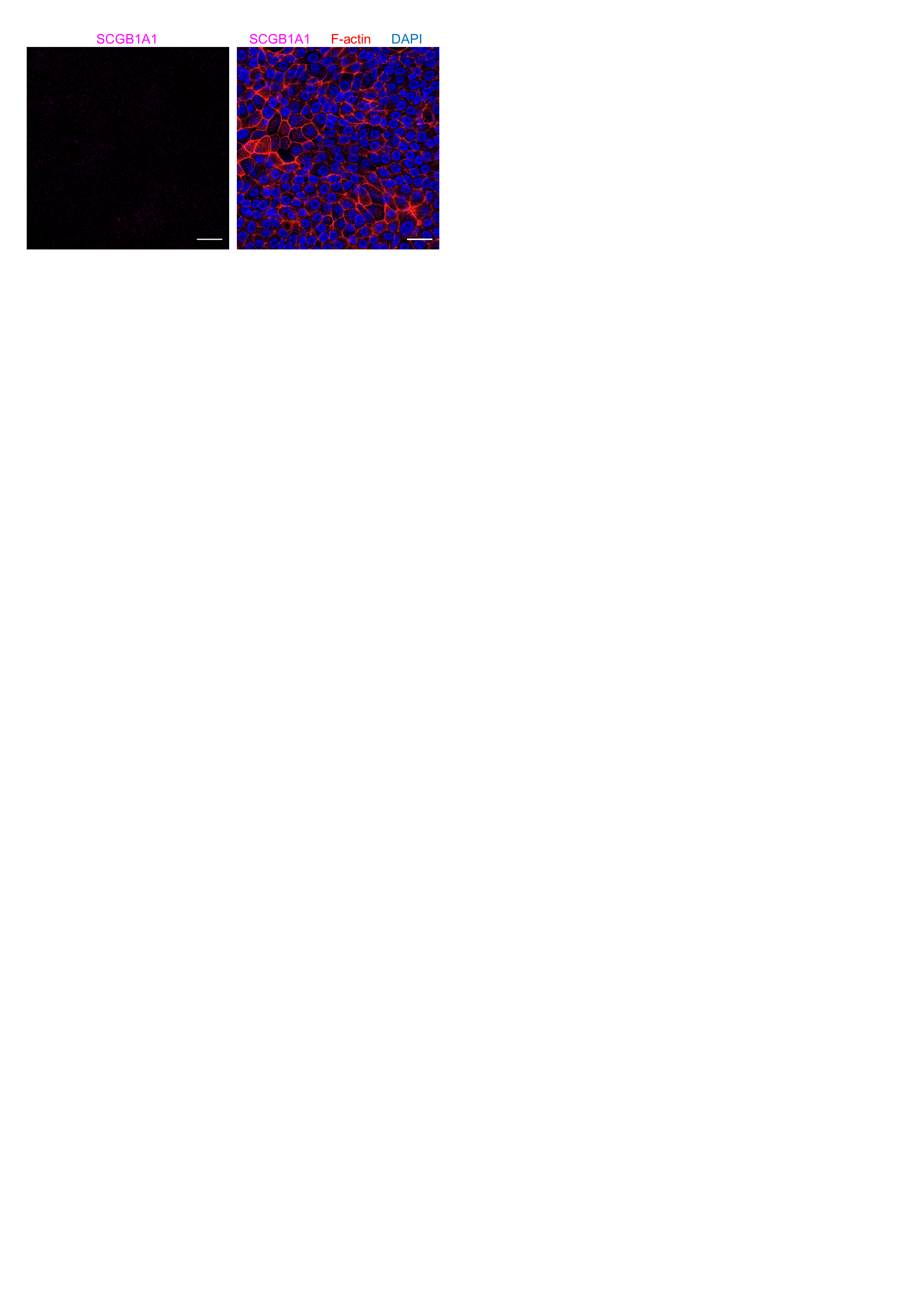

Supplement: Supplemental Material [file TEMI_A_2148561_SM6084.zip › Fig S2.tif]
