# Supplementary material for: Generation of self-replicating airway organoids from the cave nectar bat Eonycteris spelaea as a model system for studying host–pathogen interactions in the bat airway epithelium
Source: Emerg Microbes Infect. 2022 Dec 12;12(1):e2148561. doi: 10.1080/22221751.2022.2148561 (PMC9754017; doi:10.1080/22221751.2022.2148561)
Supplement: Supplementary file 2 [file TEMI_A_2148561_SM0560.docx]

**Supplementary Tables**

**Table S1. B/D expansion medium.** 

| **Name of reagent or supplement** | **Brand** | **Cat#** | **Final Concentration** |
| --- | --- | --- | --- |
| Bronchial epithelial cell medium | Sciencell | 3211 | 50% |
| Advanced (ad) DMEM/F12 | Gibco | 12634010 | 23.5% |
| R-spondin 1 conditioned medium | In-house preparation |  | 20% |
| HEPES | Gibco | 15630080 | 10 mM |
| Glutamax | Invitrogen | 35050-61 | 2.0 mM |
| P/S | Gibco | 15070-063 | 100 U/ml |
| Primicon | InvivoGen | Ant-pm-2 | 100 U/ml |
| B27 | Gibco | 12587010 | 2% |
| Hydrocortisone | Sigma | H0888 | 0.5 μg/ml |
| 3,3’,5-Triido-L-thyronine (T3) | Sigma | T6397 | 100 nm |
| Epinephrine | Sigma | E4642 | 0.5 μg/ml |
| N-acetyl-cysteine | Sigma | A9165 | 1.25 mM |
| Nicotinamide | Sigma | N0636 | 5 mM |
| TGFb inhibitor (A83-01) | Tocris | 2939 | 1 μM |
| BMPi (DMH-1) | Selleckem | S7146 | 1 μM |
| Rocki (Y-27632) | Abmole bioscience | Y-27632 | 5 μM |
| SB202190 | Sigma | S7067 | 500 nM |
| Fibroblast growth factor 10 (FGF10) | Peprotech | 100-26 | 100 ng/ml |
| Fibroblast growth factor 7 (FGF7) | Peprotech | 100-19 | 25 ng/ml |
| Insulin-like growth factor-1 (IGF-1) | Peprotech | 100-11 | 25 ng/ml |
| N-[N-(3,5-Difluorophenacetyl)-L-alanyl]-S-phenylglycine t-butyl ester (DAPT) | StemCell technologies | 72082 | 5 μM |

**Table S2. ALI-Differentiation (ALI-Diff) medium.** 

| **Name of reagent or supplement** | **Brand** | **Cat#** | **Final Concentration** |
| --- | --- | --- | --- |
| Advanced (ad) DMEM/F12 | Gibco | 12634-010 | 1x |
| Hydrocortisone | Sigma | H088 | 0.5 μg/ml |
| 3,3’,5-Triido-L-thyronine (T3) | Sigma | T6397 | 100 nM |
| Epinephrine | Sigma | E4642-5g | 0.5 μg/ml |
| hEGF | ThermoFisher | PHG0311 | 0.5 ng/ml |
| TTNPB | Cayman | No.16144 | 100 nM |
| A83-01 | Tocris | 2939/10 | 100 nM |
| Y-27632 dihydrochloride | Tocris | 1254 | 5 μM |
| P/S | Gibco | 15070063 | 100 U/ml |

**Table S3. Airway Organoid (AO) medium.** 

| **Name of reagent or supplement** | **Brand** | **Cat#** | **Final Concentration** |
| --- | --- | --- | --- |
| R-spondin 1 conditioned medium | In-house preparation |  | 25% or 0% |
| FGF7 | Peprotech | 100-19 | 25 ng/ml or 5 ng/ml |
| FGF10 | Preptotech | 100-26 | 100 ng/ml or 25 ng/ml |
| A83-01 | Tocris | 2939 | 500 nM |
| Y-27632 | Abmole | Y-27632 | 5 μM |
| SB202190 | Sigma | S7067 | 500 nM |
| B27 | Gibco | 17504-44 | 1x |
| N-acetyl-cysteine | Sigma | A9165-5g | 1.25 mM |
| Nicotinamide | Sigma | N0636 | 5 mM |
| GlutaMax | Invitrogen | 12634-034 | 1x |
| HEPES | Invitrogen | 15630-056 | 10 mM |
| P/S | Gibco | 15070-063 | 100 U/ml |
| Primocin | InvivoGen | Ant-pm-1 | 50 μg/ml |
| Advanced (ad) DMEM/F12 | Invitrogen | 12634-034 | 1x |

**Table S4: Real-time PCR primer sequences for quantifying expression of indicated** ***E. spelaea*** **genes**

| **Primer name** | **Sequence** |
| --- | --- |
| **SNRPD3 F** | TGGGAAGCTCATTGAGGCAG |
| **SNRPD3 R** | TGCCACGGATGTATACCTGC |
| **β actin F** | GTCTTTCCCTCCATCGTGGG |
| **β actin R** | TCGATGGGGTACTTCAGGGT |
| **RPL4 F** | ACCAAGGAGGCTGTTTTGCT |
| **RPL4 R** | CTCATTTTGCCCTTGCCAGC |
| **MLF1 F** | CATCTCAAACCCGACAGGCT |
| **MLF1 R** | ACGTGAGCTCGGTCATGAAG |
| **TP63 F** | CTCGGACCTGAGTGACCCTA |
| **TP63 R** | GCGCATGGTCTGTGTTGTAC |
| **FOXJ1 F** | CTACGCTACGCTCATCTGCA |
| **FOXJ1 R** | ATGGAATTCTGCCAGGTGGG |
| **DLK2 F** | CCCGCTGTGAGGTGAATGTA |
| **DLK2 R** | GGCACAGTCATCCAGGTTGA |
| **MUC5AC F** | TTCGTCATCGCCCAGACAAA |
| **MUC5AC R** | TTGAAGTTCCCGCAGAGACC |
| **BPIFB1 F** | GAAAGGCTGACACAGGAGCT |
| **BPIFB1 R** | GTTCACCAGGTTGCCCAGTA |

**Table S5: Amino acid comparison (displayed as % similarity / % identity) of homologs for key airway epithelial cell type markers and genes in human, mouse and cave nectar bats.** 

| **Cell marker** | **Human** | **Mouse** | **Cave nectar bat** |
| --- | --- | --- | --- |
| **β actin** | 100/100 | 100/100 | 100/100 |
| **TP63** | 100/100 | 99/98 | 85/83 |
| **DLK2** | 100/100 | 95/92 | 96/93 |
| **KRT5** | 100/100 | 94/90 | 99/96 |
| **KRT15** | 100/100 | 88/84 | 88/85 |
| **SCGB1A1** | 100/100 | 80/56 | 73/51 |
| **MUC5AC** | 100/100 | 78/67 | 77/69 |
| **MUC5B** | 100/100 | 72/61 | 86/77 |
| **CCDC153** | 100/100 | 83/70 | 86/76 |
| **MLF1** | 100/100 | 90/80 | 89/81 |
| **PLK4** | 100/100 | 86/79 | 92/97 |
| **CFTR** | 100/100 | 89/78 | 96/92 |
| **IL-13** | 100/100 | 59/71 | 67/76 |
